# Performance of spatial capture-recapture models with repurposed data: Assessing estimator robustness for retrospective applications

**DOI:** 10.1371/journal.pone.0236978

**Published:** 2020-08-14

**Authors:** Jennifer B. Smith, Bryan S. Stevens, Dwayne R. Etter, David M. Williams

**Affiliations:** 1 Department of Fisheries and Wildlife, Boone and Crockett Quantitative Wildlife Center, Michigan State University, East Lansing, Michigan, United States of America; 2 Department of Fish and Wildlife Sciences, Idaho Cooperative Fish and Wildlife Research Unit, University of Idaho, Moscow, Idaho, United States of America; 3 Wildlife Division, Michigan Department of Natural Resources, Lansing, Michigan, United States of America; Texas State University, UNITED STATES

## Abstract

Advancements in statistical ecology offer the opportunity to gain further inferences from existing data with minimal financial cost. Spatial capture-recapture (SCR) models extend traditional capture-recapture models to incorporate spatial position of capture and enable direct estimation of animal densities across a region of interest. The additional inferences provided are both ecologically interesting and valuable for decision making, which has resulted in traditional capture-recapture data being repurposed using SCR. Yet, many capture-recapture studies were not designed for SCR and the limitations of repurposing data from such studies are rarely assessed in practice. We used simulation to evaluate the robustness of SCR for retrospectively estimating large mammal densities over a variety of scenarios using repurposed capture-recapture data collected by an asymmetrical sampling grid and covering a broad spatial extent in a heterogenous landscape. We found performance of SCR models fit using repurposed data simulated from the existing grid was not robust, but instead bias and precision of density estimates varied considerably among simulations scenarios. For example, while the smallest relatives bias of density estimates was 3%, it ranged by 14 orders of magnitude among scenarios and was most strongly influenced by detection parameters. Our results caution against the casual repurposing of non-spatial capture-recapture data using SCR and demonstrate the importance of using simulation to assessing model performance during retrospective applications.

## Introduction

Applied ecological research must balance the pursuit of accurate inference against the logistical and financial constraints that accompany study of organisms in natural field settings. Technological and analytical developments offer potential relief from these constraints by extending the ecological inferences possible from data collected under existing study designs. As a consequence, researchers often repurpose their data (i.e., use them for a purpose they were not originally intended for) to take advantage of methodological advancements after data collection has occurred [1–4], presumably allowing for improved inferences from data that already exist. Data repurposing of this nature will undoubtedly become more common in the future as methods continue their rapid proliferation in statistical ecology [5]. Such repurposing is especially valuable given that methodological developments often allow us to advance our understanding of the magnitude of ecological states and processes that are directly informative for management and conservation (e.g., the size and distribution of animal populations; [6–8]).

Accurate estimates of animal abundance are a fundamental component of effective decision making in conservation and natural resource management [7]. A variety of population-estimation methods have been employed in applied ecology, and these tools commonly rely on harvest, point-count, distance, or capture-mark-recapture (CMR) data [7,9–12]. CMR studies are particularly useful for estimating animal abundance, and the statistical models arising from CMR have been continually refined to improve their applicability to different data types [13–17]. However, classical CMR models do not explicitly estimate the effective area sampled by their capture methods and thus cannot directly estimate animal density without additional assumptions or auxiliary information. Density is a valuable metric for management and conservation and, consequently, researchers employing CMR studies often estimate density by imposing either an ad-hoc spatial extent or using additional data (such as telemetry) to delineate an effective sampling area for their capture methods [18].

Spatial capture-recapture (SCR) models are a relatively new extension of CMR models that use information on the location of capture to explicitly estimate the density of animal activity centers, whereby animal abundance is a derived parameter [19,20]. The advent of SCR has created an opportunity to extract valuable spatially explicit estimates of animal abundance and density from existing CMR datasets that were not collected with SCR in mind. In addition to recaptures of individual animals (a required component of CMR models), estimation of abundance using SCR requires detection of unique individuals at multiple locations in space (i.e., spatial recaptures). In the absence of a sufficient number of spatial recaptures it becomes difficult to estimate parameters of the detection process, and consequently challenging to generate reliable estimates of density using SCR [21]. The ability of any CMR study to generate spatial recaptures is affected by the spatial configuration of capture devices relative to movement patterns of the animals under study. All CMR studies should place traps in a manner that attempts to reflect the spatial distribution of their study species [22,23]. Yet, spatial recaptures are not an explicit component of conventional CMR abundance estimators (i.e., no information on location of capture is used in abundance estimation), and therefore the prioritization of spatial recaptures was not an emphasis of study design [22,23]. This potential limitation of historical CMR data manifests itself conspicuously for large-scale monitoring studies that faced tradeoffs between maintenance of sufficient spatial coverage of the trapping grid and the reasonable spacing and arrangement of individual traps [24]. As a consequence of these tradeoffs and the relative newness of SCR it seems unlikely that many large-scale monitoring programs initiated prior to the dissemination of SCR theory prioritized the recapture of individual organisms at multiple locations in space (i.e., prior to 2008; [19, 20]).

Despite the subtle differences in data and study design requirements, some studies have repurposed data collected for non-spatial CMR analyses using SCR models (e.g., [25]). While this practice may provide useful information on animal abundance, such retrospective data repurposing assumes that the original study provided the additional information required to make reliable inference about density using SCR models, and such assumptions are rarely assessed in practice. Previous studies have often assessed the adequacy of SCR models by comparing the similarity of density estimates generated via SCR and ad-hoc methods using existing CMR data—a situation in which the distance between either estimate and truth is unknown [26–28]. Conversely, many studies have used simulation to proactively inform the design of future SCR studies by assessing performance of potential study designs [21,24,25,29–31]. However, we are unaware of studies that used simulation to retrospectively evaluate robustness of SCR estimates over a range of plausible conditions (e.g., true population densities, detection parameters, etc.) when true density is unknown and field data from a large-scale, non-spatial CMR monitoring program are being repurposed to generate estimates (and hence the sampling grid is fixed prior to analyses). As such, the robustness of estimates from SCR models that used repurposed CMR data collected from large-scale monitoring programs with fixed sampling grids is incompletely understood.

Our objective was to assess the robustness of performance of SCR for estimating density using data repurposed from a CMR study that used a fixed sampling grid, which was designed prior to the development of SCR. We used a case-study of population monitoring for American black bears (*Ursus americanus*) in the northern Lower Peninsula (NLP) of Michigan, where noninvasive hair-snare data was collected for CMR population estimation at the spatial extent of the entire NLP [32]. Though this sampling effort resulted in a grid that was asymmetric due to heterogeneous land cover and land ownership patterns, independent analyses of this population indicated that abundance estimates produced by CMR were reasonable [33,34]. We used stochastic simulation to evaluate robustness of SCR density estimators under a variety of plausible true parameter combinations. While our case study focused on black bear population monitoring in Michigan, USA, our findings have relevance and implications for other studies seeking to repurpose traditional CMR monitoring data collected over large scales.

## Methods

### Data and model description

**Fig 1:**
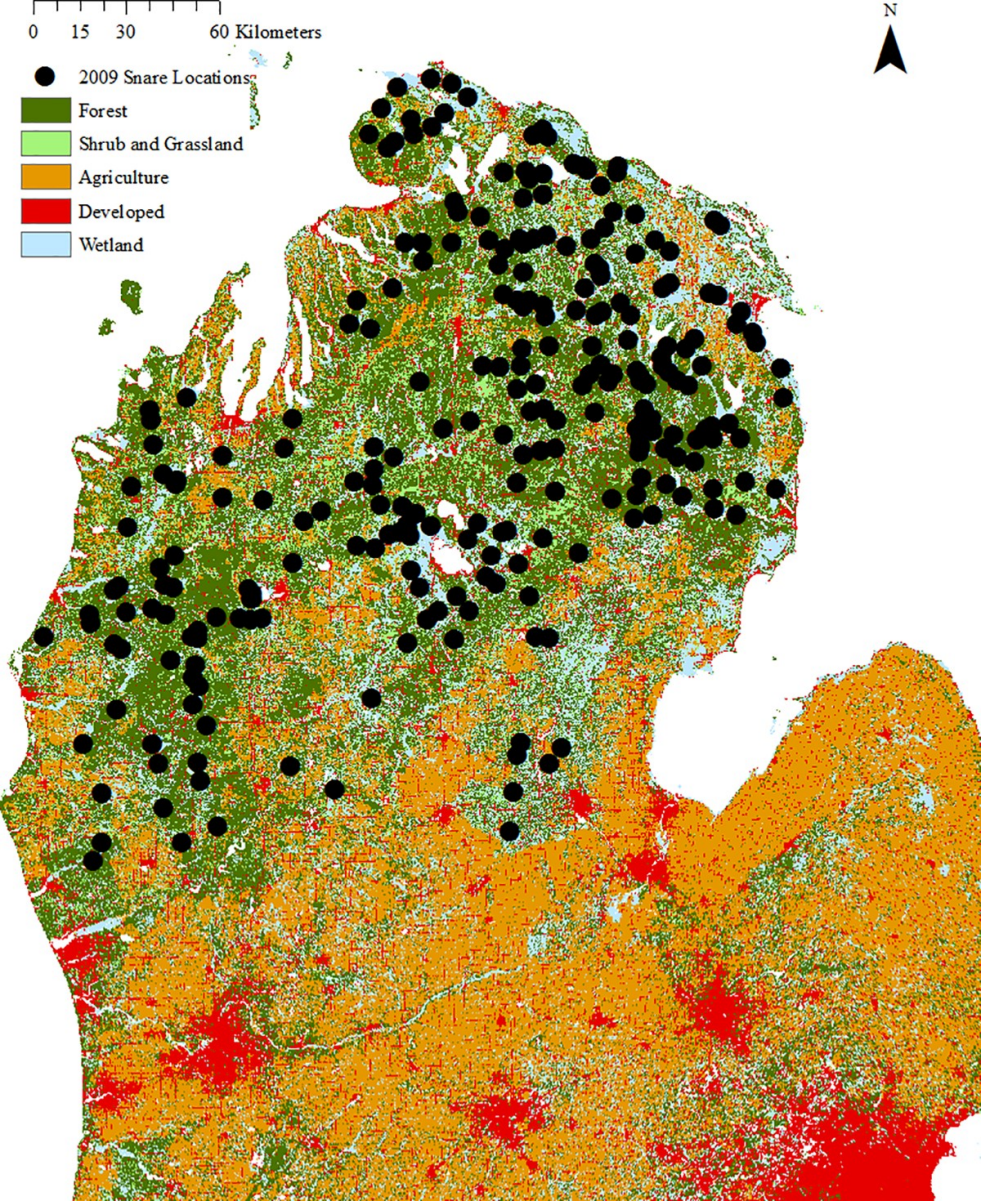
Hair snare trap locations used in simulations. Locations reflect placement in 2009 in the northern Lower Peninsula of Michigan. These locations were constrained by patchy habitat configuration and heterogeneous land ownership. Land cover imagery source: Map services and data available from U.S. Geological Survey, National Geospatial Program.

We simulated CMR data from an existing array of barbed-wire hair snares in the NLP of Michigan. The snare array was used to estimate abundance of black bears in the NLP as part of a population monitoring program initiated by the Michigan Department of Natural Resources in 2003. The trapping array consisted of 257 snares distributed across 36,848 km^2^ in a spatially irregular but locally clustered design (Fig 1). Average nearest-neighbor distance between traps was 5.8 km (SD = 3.5 km, range = 0.48–20.9 km); this average spacing is similar to the range of other black bear hair-snare arrays [24,25,35], but the range of distances is likely wider. The placement of traps across the spatial extent of the study area was necessarily irregular and affected by both the structure of land cover and patterns of land ownership. Specifically, the region consists of fragmented forests with roads, human development, and agriculture, and exists as a mosaic of public and private land ownership. Consequently, the preferred, regular trapping grids that are commonly evaluated in simulation studies (e.g., [25]) were not feasible for this study region and spatial extent. The spatial extent of the trapping array was defined by the boundaries of three bear management units in the NLP used by Michigan Department of Natural Resources, which encompass the latitudinal area between Mackinaw City and Muskegon, MI (Fig 1). Geographic coordinates of the snare locations are available in S5 Appendix.

Despite the irregular and large-scale nature of the study design, abundance estimates from the original CMR study have been externally validated as reasonable by studies that estimated the effective population size using genetic analysis [33] and estimated the population vital rates from a statistical catch-at-age analysis [34]. Furthermore, the population estimated by the CMR data was approximately closed (spatially and demographically) due to the geographical features of the NLP and the timing of the study relative to bears’ hibernation and the hunting season. Specifically, the extent of the array was bounded by Lake Michigan to the west, Lake Huron to the east, the Straights of Mackinaw to the north, and non-habitat (intensive agriculture and urban land covers) to the south [32]. Additionally, hair-snare sampling began after bear emergence from hibernation and ended before the beginning of the fall hunting season; historically, survival rates during these summer months were over 96%, suggesting demographic closure assumptions were reasonable [36]. To mimic past sampling and maintain this assumption of demographic closure, our simulations were constrained to sample over a maximum number of 7 weeks.

We simulated performance of SCR models for estimating black bear density from capture data randomly generated from the existing array of hair snares in the NLP. SCR methods model the distribution of animals as a spatial point process, and the probability of detecting an individual animal at a specific trap is a function of the distance between the trap and the animal’s activity center [19,37]. The probability of detection when distance equals zero is represented by the parameter *g*_0_, whereas the shape of the detection function is determined by *σ*, which dictates the rate at which detection probability decreases (from a maximum of *g*_0_) as distance from the trap to the activity center increases. We used a halfnormal detection function $p=g_{0}\;\exp\;(\frac{{-d}^{2}}{{2\sigma }^{2}})$, where *p* is the probability of detection and *d* is the distance between an animal’s activity center and a trap. By incorporating geographic information about the locations of detections for individual animals, SCR models can explicitly estimate the effective sampling area of the trapping grid and directly estimate population density (*D*) as the number of animal activity centers within that area [37]. Thus, SCR models estimate three parameters to enable spatially explicit inference about animal density. Importantly, the hair-snare arrangement used to estimate bear abundance in the NLP of Michigan was designed for abundance estimation and before the development of SCR methods (circa 2003; [32]), and therefore did not specifically prioritize spatial recaptures of individual bears in its conception. Thus, our simulations generated hair snare CMR data from a non-spatial study design, which mimicked repurposing of CMR data for estimating bear density using SCR models, but under known sets of conditions.

### Simulation study design

We simulated data generation and black bear density estimation for combinations of bear density, detection parameter values, and sampling effort to assess the robustness of performance for SCR density estimates over a plausible range of conditions using the existing trapping grid. Specifically, we simulated performance over different combinations of *D*, *σ*, *g*_0_, and the number of sampling events (*k*). Data generation occurred over a four-dimensional parameter space that included 2 levels of bear density and 3 levels each of *σ*, *g*_0_, and sampling effort, for 54 total scenarios. We reviewed existing literature on black bears to determine reasonable values of model parameters and inform the simulation study design. Bear density (*D*) was simulated at 10 and 50 bears per 100 km^2^ and values of *σ* were simulated at 2, 5, and 12 km [24,38–40], while values of *g*_0_ were simulated at 0.005, 0.02, and 0.2 [25,32]. Given that researchers repurposing traditional CMR data using SCR models generally do not know the true parameter values for their study population, literature review provides a useful starting point for determining bounds of the parameter space over which it is reasonable to assess estimator robustness. Consequently, the range of parameter values considered here represents plausible parameter combinations for NLP black bear populations; therefore, assessment of estimator robustness over these conditions provides an understanding of the limitations and reliability of such estimates when repurposing data under the existing grid design. Finally, we simulated sampling efforts (*k)* that included the original data collection protocol (5 occasions, [32]), as well as shortened (3 occasions) and extended (7 occasions) sampling options to determine the influence of sampling effort on estimators.

Data from each scenario were simulated using the secrdesign package (version 2.5.2), and models were subsequently fit, and parameters estimated, using maximum likelihood via the secr package (version 3.1; Efford 2010) in R (version 3.4.1; R Core Development Team 2017). We specified the basic version of the SCR model [18] and assumed activity centers were distributed according to a homogenous Poisson point process with circular home ranges. In addition, our simulations assumed the detection model was correctly specified as a halfnormal function. For each simulation scenario we replicated data generation and parameter estimation 100 times, generating 100 detection histories and sets of parameter estimates per scenario. We monitored each scenario for error messages indicating whether any of the 100 replications experienced model convergence problems. Any replicates that failed to converge were not included in our evaluation of model performance. We evaluated bias of density estimates using the mean of the relative difference, which was calculated as the average of the relative difference between true density and each estimate ($\frac{1}{n}\sum_{i=1}^{100}\frac{\left(\hat{D}-D_{truth}\right)}{D_{truth}}$; *n* = 100). We assessed precision of estimators using the estimated coefficient of variation of the density estimates ($s_{\hat{D}}/\bar{\hat{D}}$), calculated from the simulated sampling distribution of $\hat{D}$ for each scenario.

## Results

**Fig 2:**
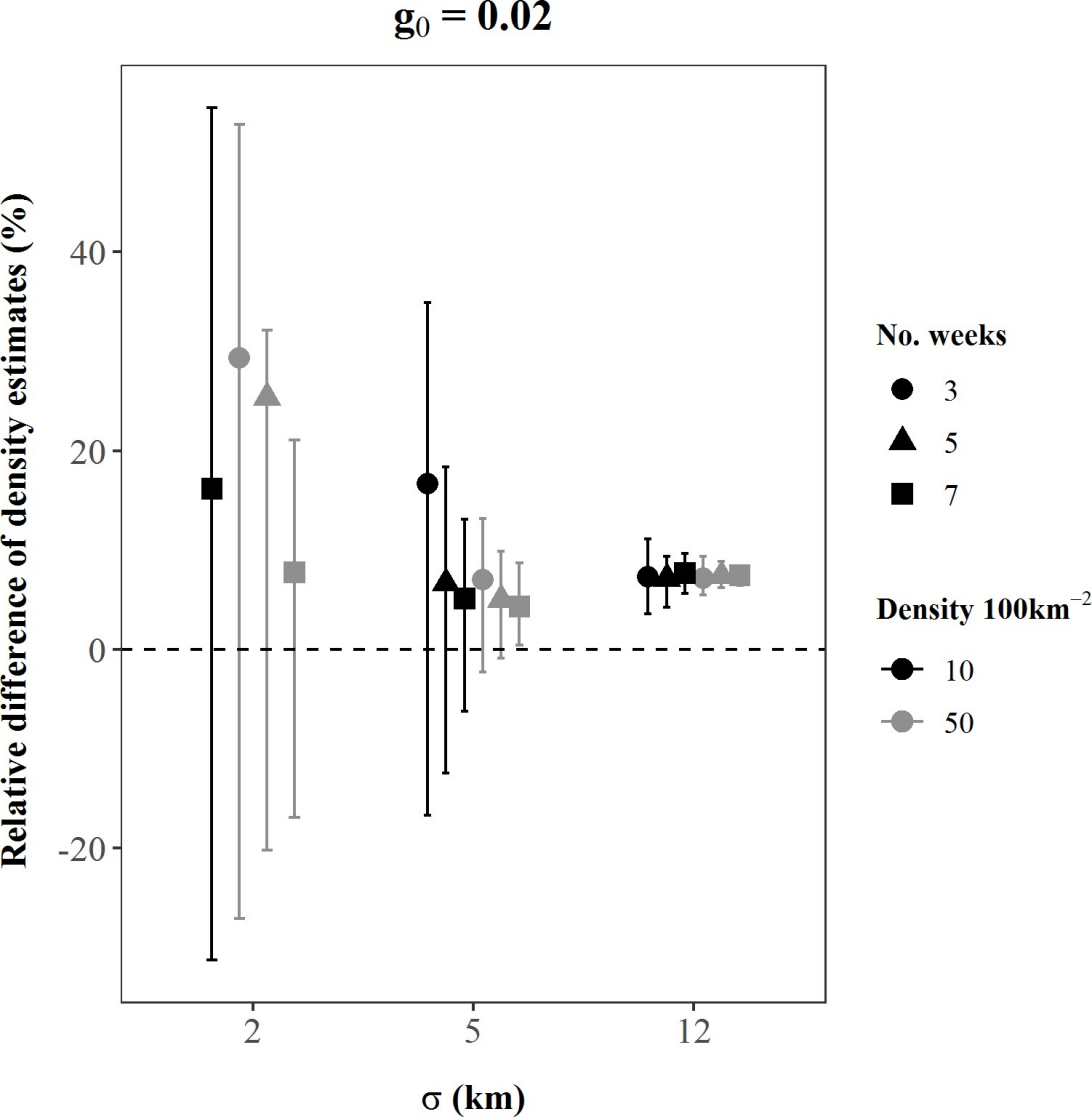
Dot and whiskers showing the mean (bias) and interquartile range of the percent relative differences of SCR density estimates from each of 18 simulated scenarios with *g*_0_ = 0.02. The x-axis values indicate the simulated *σ* (km) value for each scenario, while dot shapes identify the simulated sampling effort, *k* (weeks). Black dot and whisker elements are scenarios simulated with *D =* 10 bears/100 km^2^; grey elements are scenarios simulated with D *=* 50 bears/100 km^2^. The number of replications included in these calculations varied among scenarios because only replications that successfully converged were retained and convergence rates differed among scenarios. Some dot and whisker elements are not displayed due to extreme values of density estimates within that scenario; summary statistics of these scenarios are available in S1 Table. The dashed line indicates the estimate and true density were equal (relative difference = 0).

**Fig 3:**
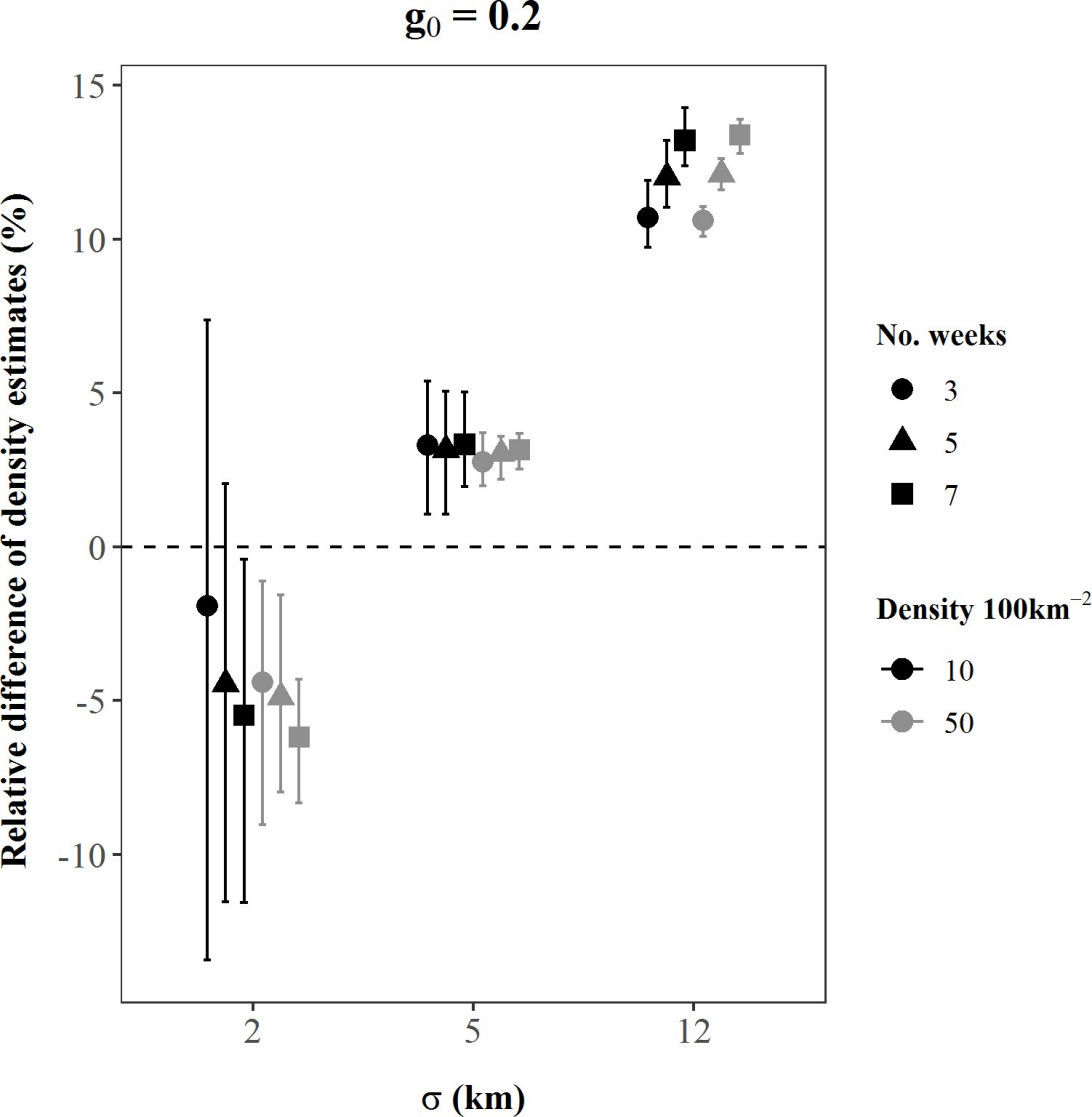
Dot and whiskers showing the mean (bias) and interquartile range of the percent relative differences of SCR density estimates from each of 18 simulated scenarios with *g*_0_ = 0.2. The x-axis values indicate the simulated *σ* (km) value for each scenario, while dot shapes identify the simulated sampling effort, *k* (weeks). Black dot and whisker elements are scenarios simulated with *D =* 10 bears/100 km^2^; grey elements are scenarios simulated with D *=* 50 bears/100 km^2^. The number of replications included in these calculations varied among scenarios because only replications that successfully converged were retained and convergence rates differed among scenarios. The dashed line indicates the estimate and true density were equal (relative difference = 0).

**Fig 4:**
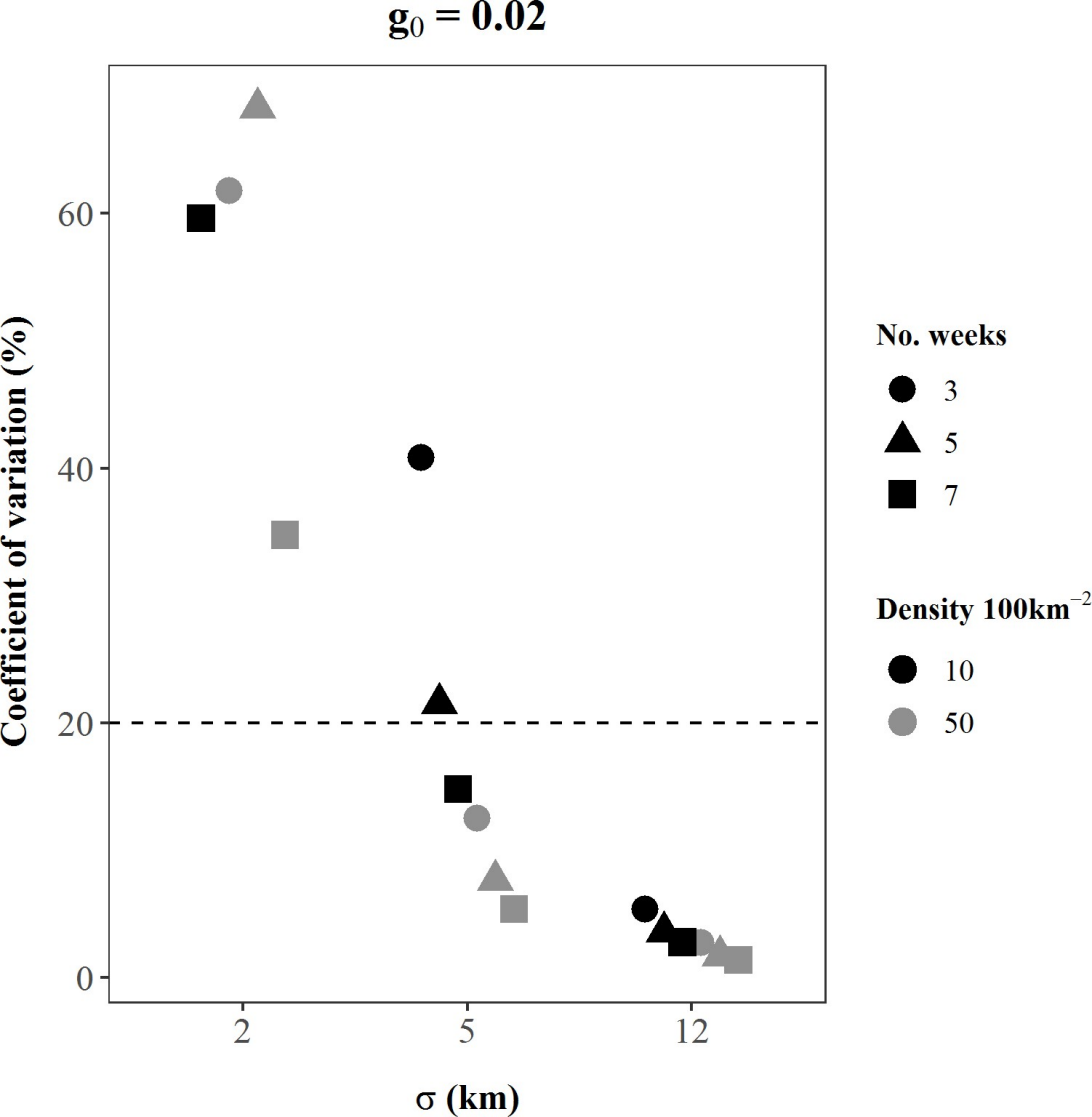
The percent coefficient of variation of SCR density estimates from each of 18 simulated scenarios with *g*_0_ = 0.02. The x-axis values indicate the simulated *σ* (km) value for each scenario, while dot shapes identify the simulated sampling effort, *k* (weeks). Black symbols are scenarios simulated with *D =* 10 bears/100 km^2^; grey symbols are scenarios simulated with D *=* 50 bears/100 km^2^. Coefficient of variation values for 2 scenarios are not displayed because they were extreme outlier values. Summary statistics of these scenarios, including coefficient of variation values, are available in S1 Table. The number of replications included in these calculations varied among scenarios because only replications that successfully converged were retained and convergence rates differed among scenarios. The dashed line indicates CV = 20%, which is the recommended coefficient of variation value for reasonable precision of estimates [23].

**Fig 5:**
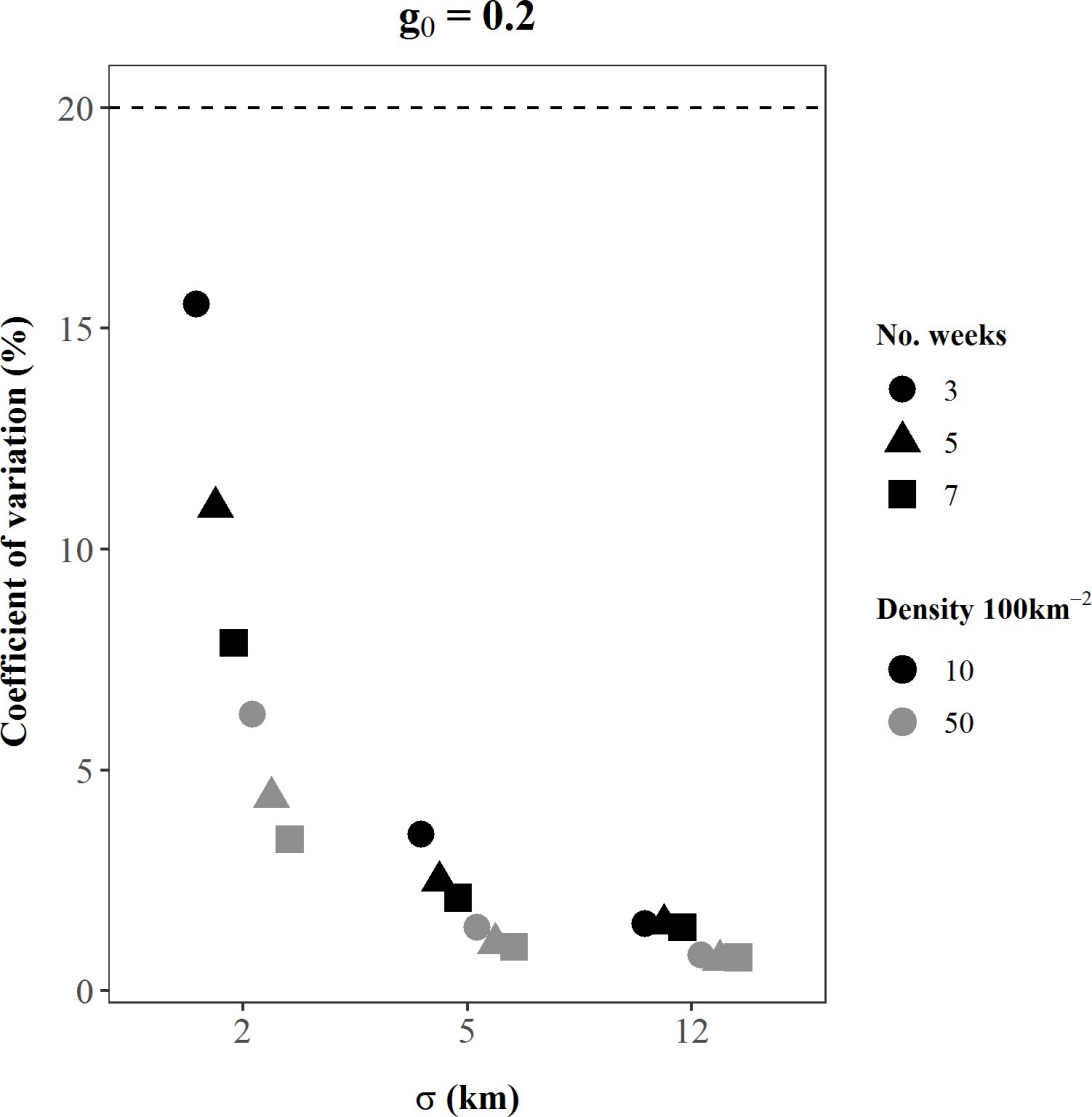
The percent coefficient of variation of SCR density estimates from each of 18 simulated scenarios with *g*_0_ = 0.2. The x-axis values indicate the simulated *σ* (km) value for each scenario, while dot shapes identify the simulated sampling effort, *k* (weeks). Black symbols are scenarios simulated with *D =* 10 bears/100 km^2^; grey symbols are scenarios simulated with D *=* 50 bears/100 km^2^. The number of replications included in these calculations varied among scenarios because only replications that successfully converged were retained and convergence rates differed among scenarios. The dashed line indicates CV = 20%, which is the recommended coefficient of variation value for reasonable precision of estimates [23].

Performance of SCR density estimators under the existing study design was not robust across plausible values of model parameters represented by our simulation scenarios. The absolute value of bias of bear density estimates ranged over 14 orders of magnitude among scenarios, from 0.03–6.6 x 10^12^, and the coefficient of variation of density estimates ranged from 1–975%; only 6% of our simulated density estimates were < 5% biased and only half of all scenarios had a CV < 10% (Table 1). In addition, 74% of scenarios experienced convergence problems, where ≥ 1 simulation iteration either did not converge due to a likelihood maximization error or could not calculate variances of the parameter estimates (Table 1). Bias and precision of density estimates were affected by all three model parameters, as well as sampling effort. As the magnitude of the parameters and sampling effort increased, accuracy often improved (Figs 2–5). For instance, in scenarios with medium *g*_0_ and *σ* values, as bear density and sampling effort increased, bias reduced by up to 60% (Fig 2) and CV reduced by up to 70% (Fig 4; Table 1). Similarly, bias declined, and precision improved as σ increased. These findings were generally consistent among small *g*_0_ scenarios (S1 Table), and among medium *g*_0_ scenarios when *σ* was held to small or medium values (Figs 2 and 4). However, these patterns were not consistent at large values of the detection parameters, as the influence of *σ* and *g*_0_ became dominant over the effects of increased *D* and *k* (Figs 2–5).

**Table 1:** Percent bias (mean relative difference) and coefficient of variation of 100 density estimates from a SCR model, and number of failed replications (out of 100) for 54 simulated scenarios.

*g*_0_	σ (km)	D (100km^-2^)	k (wks.)	Bias (%)	CV (%)	No. failed replications
0.005	2	10	3	6.62E+14	360	81
0.005	2	10	5	7.02E+12	284	78
0.005	2	10	7	4.73E+14	547	70
0.005	2	50	3	4.25E+14	465	71
0.005	2	50	5	2.34E+12	663	56
0.005	2	50	7	412696	854	27
0.005	5	10	3	2.64E+11	736	36
0.005	5	10	5	74	89	9
0.005	5	10	7	2.23E+13	975	5
0.005	5	50	3	56	86	9
0.005	5	50	5	25	56	2
0.005	5	50	7	13	29	2
0.005	12	10	3	15	25	1
0.005	12	10	5	9	15	0
0.005	12	10	7	7	10	0
0.005	12	50	3	7	10	0
0.005	12	50	5	7	7	3
0.005	12	50	7	7	4	1
0.02	2	10	3	1236652	708	47
0.02	2	10	5	1.03E+09	943	11
0.02	2	10	7	16	60	2
0.02	2	50	3	29	62	9
0.02	2	50	5	25	68	1
0.02	2	50	7	8	35	2
0.02	5	10	3	17	41	3
0.02	5	10	5	7	22	3
0.02	5	10	7	5	15	1
0.02	5	50	3	7	13	1
0.02	5	50	5	5	8	3
0.02	5	50	7	4	5	0
0.02	12	10	3	7	5	1
0.02	12	10	5	7	4	5
0.02	12	10	7	8	3	2
0.02	12	50	3	7	3	4
0.02	12	50	5	7	2	6
0.02	12	50	7	7	1	7
0.2	2	10	3	-2	16	0
0.2	2	10	5	-4	11	0
0.2	2	10	7	-5	8	0
0.2	2	50	3	-4	6	0
0.2	2	50	5	-5	4	0
0.2	2	50	7	-6	3	1
0.2	5	10	3	3	4	1
0.2	5	10	5	3	2	0
0.2	5	10	7	3	2	1
0.2	5	50	3	3	1	5
0.2	5	50	5	3	1	4
0.2	5	50	7	3	1	3
0.2	12	10	3	11	2	16
0.2	12	10	5	12	2	0
0.2	12	10	7	13	1	0
0.2	12	50	3	11	1	14
0.2	12	50	5	12	1	0
0.2	12	50	7	13	1	0

Each scenario was defined by the combined values of 3 model parameters (*σ*, *g*_0_, and *D*) and *k*, the number of weeks sampled.

The parameters of the detection process (*σ* and *g*_0_) governed the behavior of bear density estimates more strongly than *D* or *k*. When *σ* or *g*_0_ were simulated at large values (individually or in concert), the influence of *D* and *k* on bias was typically negligible or nonexistent (Figs 2 and 3), and the influence of *D* and *k* on precision was also notably less (Figs 4 and 5). Moreover, bias and precision did not always improve when *σ* and *g*_0_ were at medium-high values and dominated the behavior of density estimates. Instead, density estimates were often very precise but biased, and *g*_0_ and *σ* interacted to determine the magnitude and direction of bias (Figs 2 and 4; Figs 3 and 5). Scenarios with large *g*_0_ and medium *σ* produced precise estimates (average CV < 2%, Table 1) with low bias (3%, Table 1), regardless of the value of *D* or *k*. However, when *σ* increased to large values and *g*_0_ was also large, density estimates remained precise, but mean bias was positive at 11–13% (Fig 3).

## Discussion

Since their development, use of SCR models for density estimation has become increasingly widespread. The utility of these methods is exciting, yet their application has arguably outpaced methodical testing of their performance under pragmatic field applications [31]. Repurposing previously collected CMR data into SCR models is among these pragmatic and increasingly common applications. We demonstrate that black-bear density estimates were not robust over a range of plausible true population and detection parameter values when simulated under a large-scale, non-spatial CMR design used to estimate black bear abundance in northern Michigan. Instead, the expected performance of estimators changed strongly with the values of model parameters that are generally unknown to researchers when they are repurposing CMR field data. Moreover, our findings reveal that the model parameters interacted to determine the expected performance of density estimators. Although not all CMR data will be poorly suited for SCR analyses, we demonstrate that a snare array which previously yielded reasonable CMR abundance estimates [33,34] can produce biased and imprecise estimates of density using SCR under a wide range of plausible conditions. Consequently, we urge caution when repurposing existing CMR data that were not collected for density estimation via SCR, even if those CMR data produced reliable abundance estimates, and emphasize the importance of critically evaluating the suitability of existing study designs before repurposing data for additional inferences.

We demonstrated that density estimates were not robust under the existing broad-scale sampling grid employed for black bears in northern Michigan. While a few simulation scenarios produced estimates with minimal bias and high precision, researchers will generally not know if data from a repurposed field study were generated under conditions where estimates were reliable. The underlying detection parameters are typically unknown, as is the density of the study organism, and inference about these quantities is often the primary motivation for applying SCR in the first place. Other recent studies have also questioned the robustness of SCR model performance in field scenarios and demonstrated the complexity of estimator performance. For example, Gerber and Parmenter ([41]) evaluated performance of SCR estimators applied to a known small mammal population and identified consistent bias in density estimates. Notably, this bias occurred despite a large sampling effort that was implemented over a small study area (~ 0.04 km^2^). While we used a different trapping design and target species, both our results and Gerber and Parmenter ([41]) demonstrate that estimates from SCR models based on repurposed field data may lack robustness (i.e., reliable estimates may only be generated under a narrow range of conditions), and reveal that estimator performance for a given grid design is sensitive to attributes of the population under study. This sensitivity is likely driven in part by our finding that true parameter values can interact to govern performance of density estimators. Such interactions complicate repurposing of data using SCR models because the behavior of the estimator is hard to generalize. Therefore, simulation of SCR performance under plausible scenarios is recommended prior to repurposing CR data, especially if it is possible that data collection occurred in the context of lower detection probabilities or sigma values.

We used simulation to retrospectively evaluate the suitability of data generated under a fixed CMR study design for building SCR models because it allowed for quantification of bias and precision of resulting density estimates over a wide range of plausible true parameter values. Previous studies that repurposed CMR data using SCR typically compared SCR density estimates to ad-hoc density estimates generated from the non-spatial CMR analyses [26,28,35]. Because truth is unknown, however, these comparisons only evaluate similarity of the estimates, rather than bias or precision. Thus, while such studies often conclude that SCR estimators are adequate, if not superior to non-spatial CMR estimators under these conditions, these conclusions lack important context because the distance between true density and either the SCR or ad-hoc density estimates is unknown. Consequently, understanding of the limitations of SCR density estimators generated using repurposed CMR data collected over large scales is ambiguous.

CMR studies for populations of wide-ranging organisms, like large mammals, require trapping arrays to cover a large spatial extent. Maintaining an array with spacing small enough to ensure both detections of individuals at multiple traps and coverage of the trapping grid over a large geographic area requires many traps and is hindered by restricted land access (either in terms of patchy habitat or ownership patterns). Thus, broad-scale studies of large mammal populations using SCR are difficult to implement because of financial and logistical constraints [25], and our study demonstrates the limitations of such challenges for black bears in Michigan. Also, because our simulations assumed a homogenous Poisson point process, the complexity of needing to accurately represent spatial changes in density with the sampling array was not a factor in our results. The model performance we observed could be exacerbated under field conditions where such heterogeneity is present, if the grid arrangement is unable to capture spatial changes in density. SCR models are generally believed to be flexible and robust to variation in array design as long as trap spacing is smaller than the average home range of the study species [21,25]. However, these conclusions were drawn from arrays with relatively regular (or clustered) trap spacing compared to the array used in our study. Such regularity is not possible over large study areas in many regions, like northern Michigan, where both the habitat and land ownership patterns are heterogeneous. In such cases, gaps and irregularities emerge in the grid because regular trapping arrays encompass non-habitat or private lands where trapping is not possible. In our study, this reality likely drove insufficient numbers of spatial recaptures, thereby limiting accurate estimation of the detection process parameters [21, 24]. Importantly, the original CMR study of black bears in northern Michigan was able to produce reasonable abundance estimates despite these challenging conditions; however, our findings demonstrate the limitations of producing reliable spatially explicit density estimates under the existing study design. To our knowledge the performance of SCR models under such irregular, but pragmatic, large-scale designs has had limited assessment via simulation.

While our simulations demonstrated a lack of robustness for density estimates, confidence can be increased by pairing auxiliary information with simulation results. We recommend simulating SCR density estimation under biologically plausible combinations of parameters for the system and sampling design of interest. Then, if performance varies strongly among scenarios, investigators can use auxiliary data to approximate where the study population lies relative to the simulated parameter space. For instance, *σ* can be converted from a home range estimate; the 95% home range radius = *σ* * sqrt (5.99) [27,35]. Approximating population density from auxiliary data is more challenging; presumably repurposing data is desirable because existing knowledge of population density is limited. However, it may be possible to approximate plausible bounds of density from harvest [34]or trail-camera data, which can be effective for estimating population sizes of species that can’t be individually identified over large spatial scales [42–46], or from ad-hoc density estimates generated from non-spatial CMR model abundance estimates. If simulation reveals performance is not robust to changes in population parameter combinations and auxiliary data are not available, we suggest that collection of these data should be a priority when repurposing traditional CMR data. Alternatively, modified study designs can be explored via simulation that may facilitate reliable future density estimation using SCR methods [21,24,25]. In our case study auxiliary data were available, which suggested CMR data collection likely occurred in a scenario with low bear density, medium *σ*, and medium sampling effort (S7 Appendix). In our simulations, that combination of parameters resulted in density estimates with a positive bias between 3 and 74% and a coefficient of variation of 2–89% (Table 1).

## Conclusions

Successful repurposing of CMR data from large-scale studies using SCR models may require a sampling design that is challenging to implement for many combinations of land access and habitat heterogeneity that are common to field studies of large mammals. Yet the perception that SCR estimators are generally robust and the advantages that spatially explicit density estimates offer for decision making may encourage application of these methods without recognition of the need to critically evaluate the suitability of existing data. This is particularly concerning for estimation of threatened, rare, or elusive populations where even small amounts of bias (such as the minimum bias observed here) could have negative consequences for management of these species. Thus while repurposing data may provide a respite from logistical and financial limitations common to ecological studies, and some CMR study designs are likely to result in accurate SCR density estimation, we warn against the casual repurposing of CMR data using SCR methods and emphasize the need to formally assess estimator performance under realistic field conditions and sampling designs.

## Supporting information

S1 Table Percent bias, 25^th^ and 75^th^ quantiles, interquartile range, and standard deviation of the relative differences and the coefficient of variation of density estimates from a SCR model for 20 simulated scenarios. Each scenario was defined by the combined values of 3 model parameters (*σ*, *g*_0_, and *D*) and *k*, the number of weeks sampled.
(XLSX)

S1 Appendix Trap file for SECR simulations. Contains the location of each snare and specifies 3 sampling occasions.
(TXT)

S2 Appendix Trap file for SECR simulations. Contains the location of each snare and specifies 5 sampling occasions.
(TXT)

S3 Appendix Trap file for SECR simulations. Contains the location of each snare and specifies 7 sampling occasions.
(TXT)

S4 Appendix Code for running SECR simulations. The base code used to run simulations of each scenario in the R package SECR.
(TXT)

S5 Appendix Geographic coordinates for each snare. (TXT)

S6 Appendix SECR mask for simulations. (CSV)

S7 Appendix Details of how the case study scenario was approximated based on auxiliary data. (DOCX)
