# Supplementary material for: Performance of spatial capture-recapture models with repurposed data: Assessing estimator robustness for retrospective applications
Source: PLoS One. 2020 Aug 14;15(8):e0236978. doi: 10.1371/journal.pone.0236978 (PMC7428166; doi:10.1371/journal.pone.0236978)
Supplement: S7 Appendix — (DOCX) [file pone.0236978.s008.docx]

**S8 Appendix. Details of how the case study scenario was approximated based on auxiliary data.**

We used auxiliary data to determine the most plausible combination of parameters for the population of black bears in the NLP of Michigan relative to performance of our simulations. Fifteen black bears (7 females, 8 males) were outfitted with GPS radio collars from 2011 – 2015 in the NLP. This location data was used to calculate the average 95% home range radius of male and female bears. Radii were calculated from kernel density estimation determined by a least-squares cross validation smoothing bandwidth [1]. The average 95% home range radius was 5 km for female bears, and 12 km for male bears, which convert to *σ* = 2 and 4.9 km respectively; average *σ* of all bears was 3.25 km. In 2009, non-spatial CMR models estimated an abundance of 1,500 bears over a trapping area of 36,848 km^2^ [2]; using this information, we can approximate a minimum bear density of 4 bears/100 km^2^. Finally, the traps were sampled for 5 weeks [3].

# References

1. Silverman BW. Density Estimation for Statistics and Data Analysis. London: Chapman and Hall; 1986.

2. Mayhew SL. A synthesis of bear population dynamics in Michigan. Michigan State University, East Lansing, USA; 2019.

3. Dreher BP, Winterstein SR, Scribner KT, Lukacs PM, Etter DR, Rosa GJM, et al. Noninvasive Estimation of Black Bear Abundance Incorporating Genotyping Errors and Harvested Bear. The Journal of Wildlife Management. 2007;71(8):2684–93.
